# Supplementary material for: Bottom marine heatwaves along the continental shelves of North America
Source: Nat Commun. 2023 Mar 13;14:1038. doi: 10.1038/s41467-023-36567-0 (PMC10011364; doi:10.1038/s41467-023-36567-0)
Supplement: Supplementary file 1 — Supplementary Information [file 41467_2023_36567_MOESM1_ESM.pdf]

Supplementary Information for:

Bottom marine heatwaves along the continental shelves of North America

Dillon J. Amaya[1], Michael G. Jacox[1,2], Michael A. Alexander[1], James D. Scott[1,3], Clara Deser[4], Antonietta Capotondi[1,3], and Adam S. Phillips[4]

[1]Physical Science Laboratory, Earth System Research Laboratory, National Oceanic and Atmospheric Administration

[2]Environmental Research Division, Southwest Fisheries Science Center, National Oceanic and Atmospheric Administration

[3]Cooperative Institute for Research in Environmental Sciences, University of Colorado Boulder

[4]National Center for Atmospheric Research

Corresponding author: Dillon J. Amaya, dillon.amaya@noaa.gov, 816-916-8348

Contents of this file:

Supplementary Methods

Supplementary Tables 1-4

Supplementary Figures 1-14

Supplementary Methods

Observational comparisons to GLORYS bottom temperature

In order to further assess the fidelity of GLORYS in reproducing observed bottom temperature variability around North America we have conducted a new comparison of GLORYS bottom temperature to several *in situ* measurements of long-term bottom temperature variability within the different LMEs discussed in this paper (Figs. S11-S12 and Tables S1-S2). Below is a brief description of each observational platform used in our comparisons, separated by LME. The platform, time range, sampling frequency, location, and depth of each observation is shown in Table S1. Hyperlinks with the source of each dataset are also provided in Table S1. At every location, the observed near-bottom temperature (NBT) was compared to that from the nearest GLORYS grid cell. Unless otherwise indicated, all comparisons are based on monthly mean anomalies. At each location, anomalies are based on the climatological period with the greatest overlap between a given observational record and the GLORYS record (1993-2019).

(a) Bering Sea

We compare to two moored buoys in the East Bering Sea, M5 and M8, which have been deployed and maintained in support of the Ecosystems and Fisheries-Oceanography Coordinated Investigations (EcoFOCI) project. See https://www.ecofoci.noaa.gov/ for more details on instrument specifications.

(b) Gulf of Alaska

The Gulf of Alaska Station 1 (GAK1)[1] is located at the mouth of Resurrection Bay near Seward, Alaska and is one of the longest running oceanographic timeseries in the region.

Originally part of a repeat hydrographic section beginning in 1970, GAK1 has been supplemented by a moored array of instruments since 1998. We analyze the mooring data here. For more information on instrument specifications, see http://research.cfos.uaf.edu/gak1/.

(c) California Current System (CCS)

The Newport Hydrographic Line is a repeat research survey operated by the NOAA Northwest Fisheries Science Center in collaboration with Oregon State University[2]. The Newport Line consists of eight stations, and here we compare GLORYS to the NBT estimated from Conductivity, Temperature, and Density (CTD) casts at Station 5.

(d) Gulf of Mexico (GOM)

The West End CP Mooring[3] is located ~25 km southwest of Mobile Bay, Alabama and offers a near continuous timeseries of NBT by which to compare to GLORYS. Details of the mooring configurations and aspects of the processing can be found in Coogan et al.[3].

(e) Southeast US (SEUS)

Observed data was taken from a repeat hydrographic section in the Florida Straits conducted by the NOAA Atlantic Oceanographic and Meteorological Laboratory on the R/V Walton Smith. This survey line consists of nine stations following 27˚N from Florida to Grand Bahama Island. Here, we compare to Station 0, which is closest to the Florida coast and has a bottom depth of 155m. The NBT measurements used are based on the deepest CTD measurement for each quarterly cast, however, that depth varies from cast-to-cast ranging from 121m to 153m. To compare with GLORYS, we first identify the day on which each CTD cast was conducted and

then we isolate the daily mean GLORYS 3D temperature data at the grid cell nearest to the observations on the day corresponding to the measurement. The bottom of each CTD cast falls between vertical grid cells in GLORYS. Therefore, we then linearly interpolate this daily mean GLORYS temperature profile to the depth of the deepest CTD measurement for that time step. This procedure allows for an apples-to-apples comparison between the instantaneous observations and the comparatively uniform GLORYS data. Since these observations were conducted quarterly, it was not possible to calculate monthly anomalies or bottom marine heatwave (BMHW) statistics. Instead, we show the full NBT for both the Walton Smith CTD casts and GLORYS in Fig. S12. For more details on the Florida Straits observations, see https://www.aoml.noaa.gov/phod/wbts/.

(e) Northeast US (NEUS)

Data for this LME were taken from two sources. The first is from the Martha's Vineyard Coastal Observatory (MVCO)[4], which features an underwater node fixed to a cable tethered to the seafloor ~1.5 km off the coast of Martha's Vineyard. This node has been collecting oceanographic data (including NBT) consistently since 2001. For more information on these instruments, see https://mvco.whoi.edu/. The second data source is based on a network of monitoring stations in Passamaquoddy Bay in which monthly CTD casts are conducted across 25 stations using the R/V Pandalus[5]. For our analysis, we chose to compare GLORYS to the station located at 45.00˚N, 66.8666˚W due to its relatively long record length from 1993-2019. However, at this location, the depth of the deepest CTD measurement for each cast exceeds the model bottom depth for the nearest GLORYS grid cell by 10-30m depending on the timestep. Therefore, in order to compare apples-to-apples, at each timestep, we interpolate the observed CTD profile at this station to the

GLORYS bottom depth of 44.4m. For more information on this dataset, visit https://open.canada.ca/data/en/dataset/12184962-7879-4214-aef0-b31162f04a27.

(f) Scotian Shelf

We further compare to CTD data from the Halifax Line, a repeat hydrographic survey off the eastern coast of Nova Scotia conducted ~quarterly. Our comparisons are based on data from Station 2. Similar to the CTD data from Passamaquaddy Bay, the deepest CTD measurements from the Halifax Line are routinely deeper than the bottom depth of the nearest GLORYS grid cell by 10-40m. Therefore, in order to compare apples-to-apples, at each timestep, we interpolate the observed CTD profile at Station 2 to the GLORYS bottom depth of 109.7m. Additionally, since the measurements at Station 2 were taken ~quarterly, we follow a similar processing method as used for our comparisons of ~quarterly data along the Florida coast. Namely, for each timestep, we identify the day of the Station 2 CTD cast and compare the vertically interpolated NBT of that cast to the bottom temperature from daily mean GLORYS data for the same day. We then show the full NBT data from each dataset in Fig. S12. More details on the Halifax Line Station 2 can be found at https://www.st.nmfs.noaa.gov/copepod/time-series/ca-50101/.

(g) Labrador-Newfoundland Shelf

Finally, we compare to CTD measurements at Station 27[6] in the Avalon Channel off Cape Spear, Newfoundland. This repeat survey is one of the longest hydrographic time series in Canada with near-monthly coverage since 1948. In our analysis, we compare the nearest GLORYS grid cell to the data displayed in Fig. 20 of Cyr et al.[6], which represents the average NBT from 150-175m with 175m being the seafloor. This timeseries is readily comparable to the nearest GLORYS

grid cell, which has a bottom depth of 160.7m. For more details on Station 27, see Cyr et al.[6] and https://www.st.nmfs.noaa.gov/copepod/time-series/ca-50601/.

Supplementary References

1. Danielson, S. CTD profile time series data from the GAK1 site in the Northern Gulf of Alaska, 1970-2020. (2021).
2. Huyer, A. *et al.* The Newport line off Oregon - Studies in the North East Pacific. *Progress in Oceanography* **75**, 126–160 (2007).
3. Coogan, J., Dzwonkowski, B. & Lehrter, J. Effects of Coastal Upwelling and Downwelling on Hydrographic Variability and Dissolved Oxygen in Mobile Bay. *Journal of Geophysical Research: Oceans* **124**, 791–806 (2019).
4. Cinquino, E. *et al.* Martha's Vineyard Coastal Observatory. (2021).
5. Robinson, S. Passamaquoddy Bay monthly Conductivity Temperature and Depth (CTD) sampling (1989 - 2018) - Open Government Portal. https://open.canada.ca/data/en/dataset/12184962-7879-4214-aef0-b31162f04a27.
6. Cyr, F. *et al.* Physical Oceanographic Conditions on the Newfoundland and Labrador Shelf during 2021. 52.

Supplementary Tables and Figures

Table S1 Details for each observational platform used in our comparisons to GLORYS bottom temperature. The second column indicates platform type, ranging from moorings (M), repeat hydrographic surveys (HS), and an undersea cable (USC). The third column indicates the availability and approximate sampling frequency of observations during the GLORYS record (1993-2019). The fourth column shows the approximate near-bottom depth of the measurements. The fifth column indicates the lat/lon coordinates of the measurements. The sixth column contains hyperlinks to the various data sources used to gather these observations.

Location	**Platform**	**Time (frequency)**	**Depth**	**Lat/Lon**	**Source**
Bering Sea (BS-M8)	M	07-15-2005 to 10-11-2017 (sub-daily)	~67m	62.194˚N, 174.688˚W	Link
Bering Sea (BS-M5)	M	05-03-2005 to 10-08-2018 (sub-daily)	~65m	59.911˚N, 171.731˚W	Link
G. of Alaska (GAK1)	M	03-21-1998 to 03-05-2019 (sub-daily)	~250m	59.8450˚N, 149.4667˚W	Link
CCS (Newport Line Stn. 5)	HS	Jan-1997 to Dec-2019 (biweekly)	~55m	44.6517˚N, 124.13˚W	Link
GOM (West End CP)	M	10-20-2004 to 12-12-2018 (hourly)	~20m	30.0902˚N, 88.2116˚W	Link
SEUS (Walton Smith)	HS	05-24-2001 to 10-04-2019 (~quarterly)	~135m	27.00˚N, 79.93˚W	Link
NEUS (MVCO)	USC	06-19-2001 to 09-05-2018 (sub-daily)	~12m	41.325˚N, 70.5667˚W	Link
NEUS (Passam. Bay)	HS	Jan-1993 to Jul-2018 (~monthly)	~44m	45.00˚N, 66.8666˚W	Link
Scotian (Halifax Line Stn. 2)	HS	Jan-2000 to Dec-2017 (~quarterly)	~110m	44.2669˚N, 63.3155˚W	Link
Labrador Shelf (Station 27)	HS	Jan-1993 to Dec-2019 (monthly)	~163m	47.5467˚N, 52.5867˚W	Link

Table S2 Average BMHW intensity (˚C) and duration (months) at each location shown in Fig. S11. Values are calculated based on linearly detrended and raw (e.g., not detrended) monthly anomalies. Black numbers indicate values calculated using *in situ* observations and red numbers indicate values calculated using the nearest GLORYS grid cell.

Location	Average Intensity (˚C)				Average Duration (months)			
	**Raw**		**Detrended**		**Raw**		**Detrended**	
Bering Sea (BS-M8)	1.9	1.8	1.6	1.4	2.7	3.2	2.7	2.7
Bering Sea (BS-M5)	3.0	1.8	2.3	1.6	5.3	4.0	4.0	3.2
G. of Alaska (GAK1)	0.8	0.6	0.7	0.6	3.3	2.3	2.9	3.3
CCS (Newport Line)	1.9	2.2	1.8	2.2	1.3	1.6	1.5	2.0
GOM (West End CP)	2.1	2.0	2.1	2.0	1.3	1.3	1.3	1.4
NEUS (MVCO)	2.1	2.0	2.1	1.9	1.9	1.5	1.7	1.7
NEUS (Passam. Bay)	1.7	1.4	1.5	1.2	1.6	1.6	1.4	1.5
Lab. Shelf (Station 27)	0.8	0.9	0.8	0.9	3.0	3.0	3.0	2.7

Table S3 Average BMHW intensity (˚C) for grid cells falling within different bottom depth intervals in each LME. Values are given for both raw and detrended bottom temperature data. Note that the detrended values are the gray dots in Fig. 3.

Average Intensity (˚C)	0-50m		50-100m		100-150m		150-200m		200-250m		250-300m		300-350m		350-400m	
	**Raw**	**Det**	**Raw**	**Det**	**Raw**	**Det**	**Raw**	**Det**	**Raw**	**Det**	**Raw**	**Det**	**Raw**	**Det**	**Raw**	**Det**
East Bering Sea	2.1	1.9	1.7	1.6	1.0	1.0	0.9	0.9	0.7	0.7	0.6	0.5	0.5	0.4	0.3	0.3
Gulf of Alaska	1.6	1.5	1.3	1.3	1.0	1.0	0.8	0.8	0.6	0.6	0.6	0.5	0.6	0.5	0.5	0.5
California Current	2.4	2.4	2.9	2.9	1.8	1.8	1.2	1.2	1.0	0.9	0.9	0.8	0.8	0.8	0.7	0.7
Gulf of California	2.0	2.0	3.1	3.1	2.6	2.6	1.9	1.9	1.2	1.2	1.0	0.9	0.8	0.7	0.8	0.7
Gulf of Mexico	1.6	1.5	1.6	1.5	1.7	1.6	1.8	1.7	1.7	1.6	1.6	1.4	1.5	1.5	1.5	1.4
Southeast US	1.7	1.7	1.9	1.8	2.2	2.1	2.3	2.2	1.9	1.7	1.7	1.6	1.4	1.3	1.2	1.1
Northeast US	2.3	2.2	2.4	2.2	1.9	1.7	1.6	1.3	1.6	1.3	1.7	1.4	1.7	1.3	1.5	1.3
Scotian Shelf	1.7	1.5	1.9	1.6	2.3	1.9	2.0	1.6	1.8	1.4	1.5	1.0	1.3	0.9	1.2	0.9
Labrador Shelf	1.5	1.5	1.4	1.3	1.2	1.2	1.3	1.2	1.3	1.2	1.1	1.0	0.9	0.8	0.9	0.8

Table S4 Average BMHW duration (months) for grid cells falling within different bottom depth intervals in each LME. Values are given for both raw and detrended bottom temperature data. Note that the detrended values are the gray dots in Fig. S2.

Average Duration (months)	0-50m		50-100m		100-150m		150-200m		200-250m		250-300m		300-350m		350-400m	
	**Raw**	**Det**	**Raw**	**Det**	**Raw**	**Det**	**Raw**	**Det**	**Raw**	**Det**	**Raw**	**Det**	**Raw**	**Det**	**Raw**	**Det**
East Bering Sea	2.5	2.3	2.9	2.8	3.1	3.0	2.9	2.9	3.1	3.0	3.1	2.9	3.1	2.9	3.0	2.4
Gulf of Alaska	2.3	2.2	2.9	2.7	3.2	3.0	3.1	2.9	3.0	2.8	3.2	2.9	3.0	2.8	2.5	2.3
California Current	2.9	2.7	3.4	3.3	2.7	2.7	2.3	2.3	2.2	2.2	2.1	2.0	2.0	1.9	1.8	1.8
Gulf of California	1.8	1.7	2.3	2.3	2.9	2.8	2.8	2.8	2.3	2.4	2.2	2.3	2.2	2.3	2.6	3.9
Gulf of Mexico	1.6	1.5	1.8	1.7	1.6	1.6	1.5	1.5	1.6	1.4	1.6	1,5	1.5	1.6	1.6	1.6
Southeast US	1.4	1.4	1.5	1.4	1.6	1.4	1.5	1.4	1.6	1.4	1.5	1.4	1.4	1.3	1.4	1.4
Northeast US	1.9	1.9	2.0	1.8	2.2	2.0	2.9	2.5	3.5	2.9	2.7	2.1	1.9	1.7	1.8	1.6
Scotian Shelf	1.8	1.6	2.4	2.2	2.8	2.4	3.0	2.4	3.3	2.5	3.3	2.2	4.3	2.4	4.0	2.7
Labrador Shelf	1.7	1.7	2.2	2.2	2.5	2.4	2.7	2.5	2.9	2.6	3.1	2.6	3.0	2.4	3.5	2.6

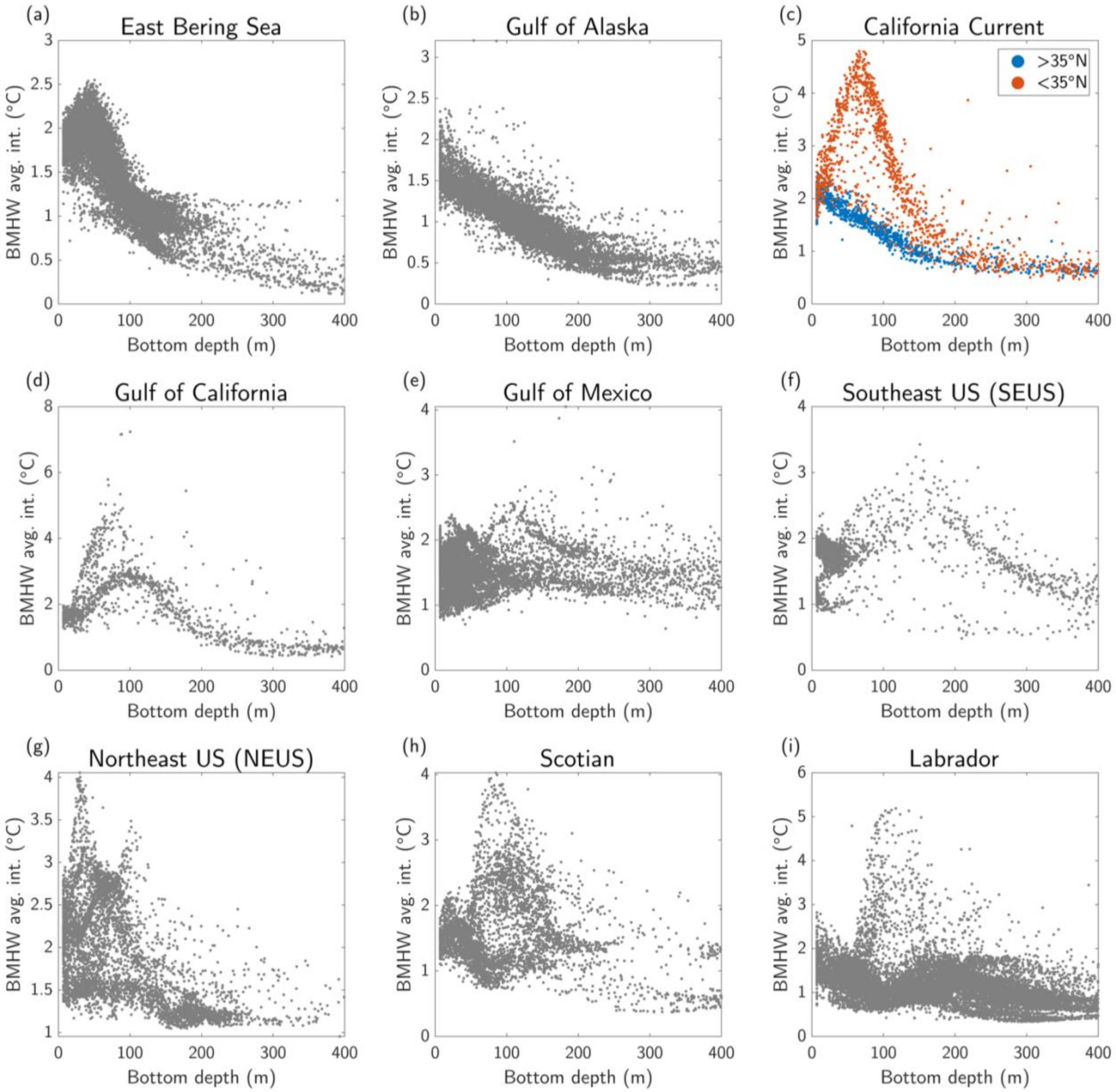

Fig. S1 Scatter plots of BMHW average intensity (˚C) versus bottom depth (m) for each grid cell in the respective LMEs. In the California Current LME (panel c), blue dots indicate grid cells north of 35˚N and orange dots indicate grid cells south of 35˚N.

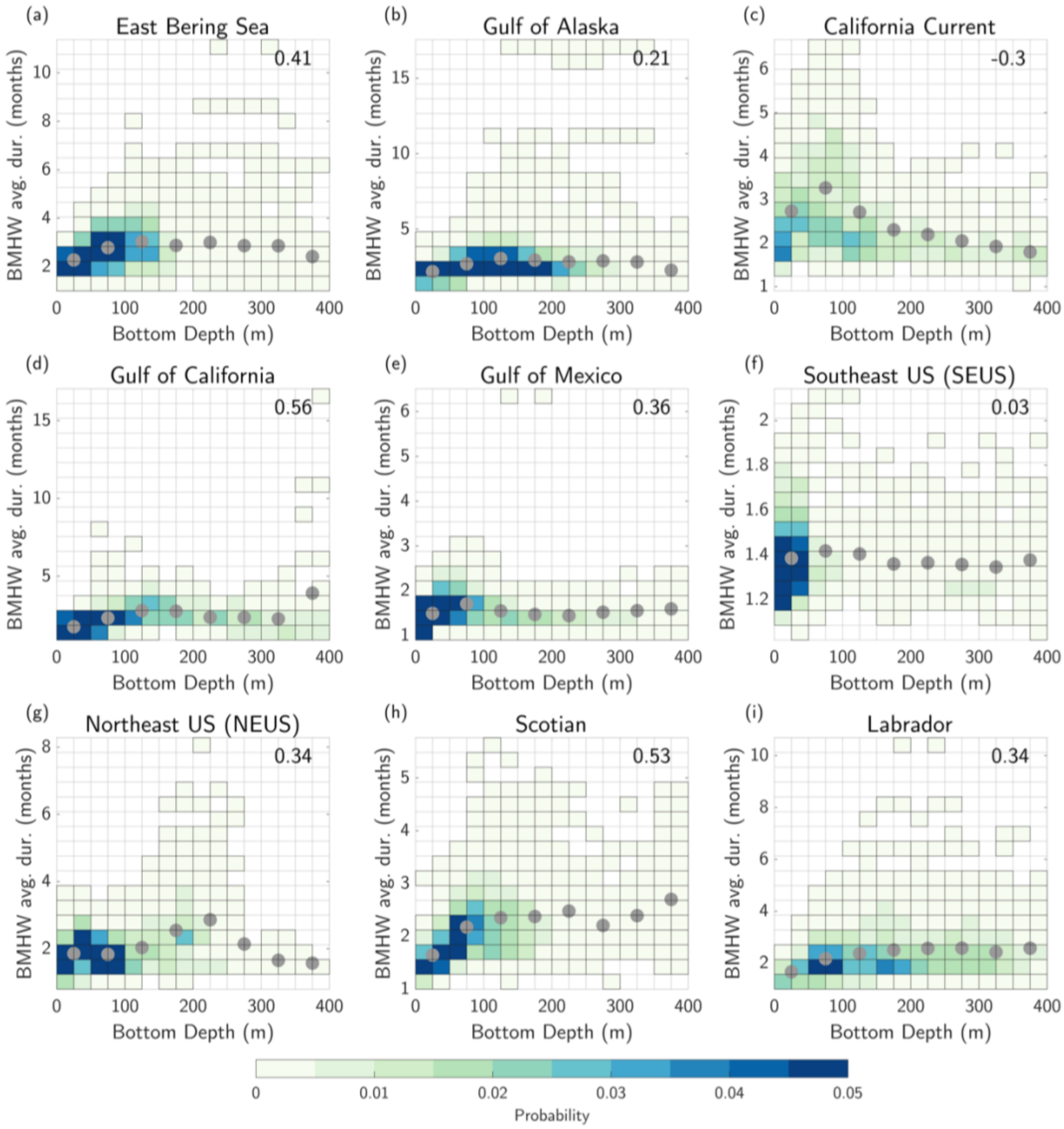

Fig. S2 Two-dimensional histograms of BMHW average duration (months) versus ocean bottom depth (m) in each LME. Shading indicates the probability that a grid cell (with bottom depth < 400m) falls within a given duration-depth interval. The Spearman correlation between BMHW average duration and bottom depth is shown in the top right of each panel. Gray dots indicate the BMHW average duration averaged across regular depth intervals of 50m. The position of each dot along the x-axis represents the center of the depth interval used for averaging. For example, the first gray dot is positioned at 25m, and represents the BMHW average duration averaged across all grid cells with bottom depths of 0-50m.

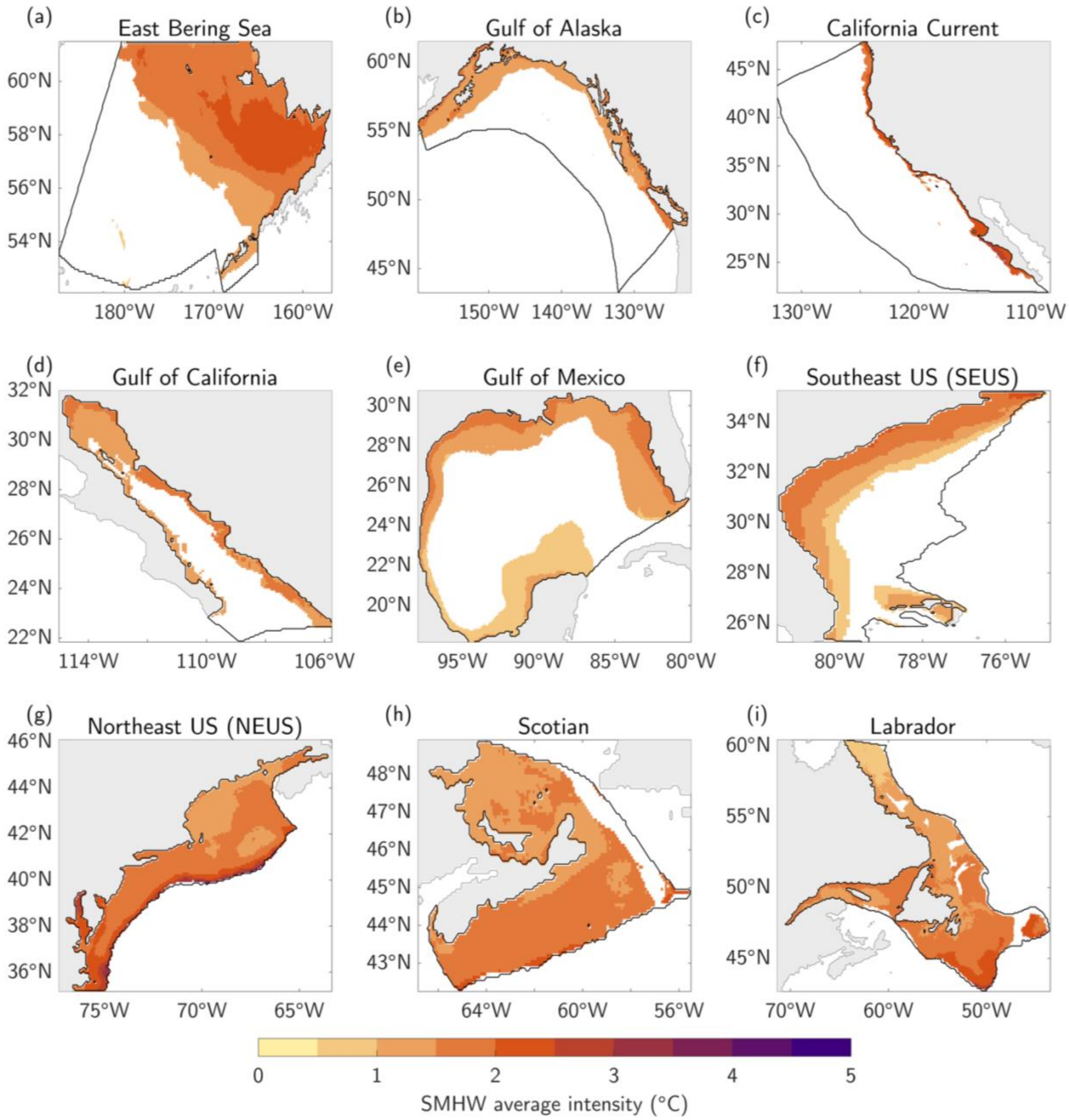

Fig. S3 Sea surface temperature anomalies (˚C) averaged during all SMHW months from 1993-2019 in each LME.

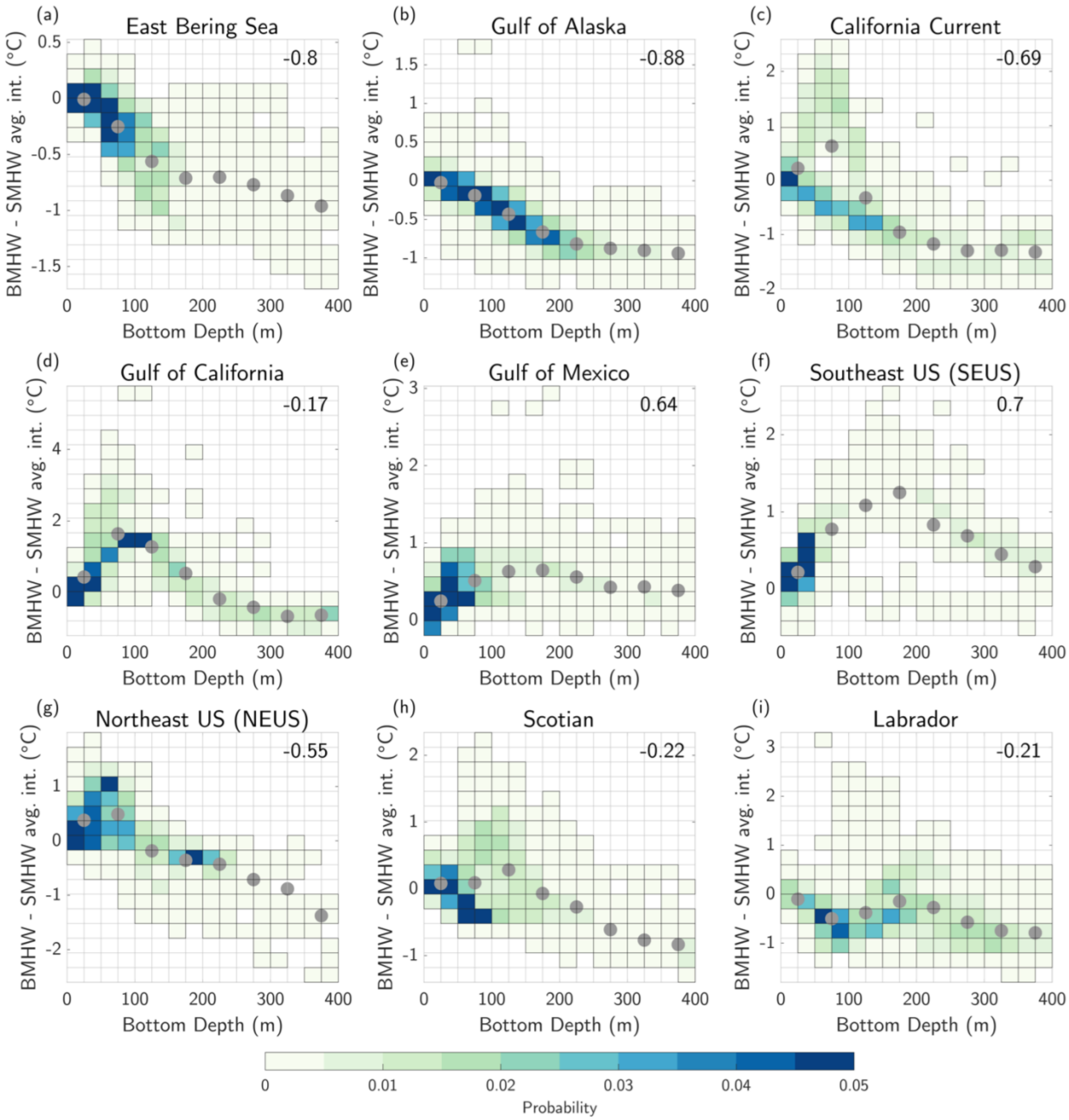

Fig. S4 As in Fig. S2, but for the difference of BMHW minus SMHW average intensity (˚C) versus bottom depth (m) in each LME.

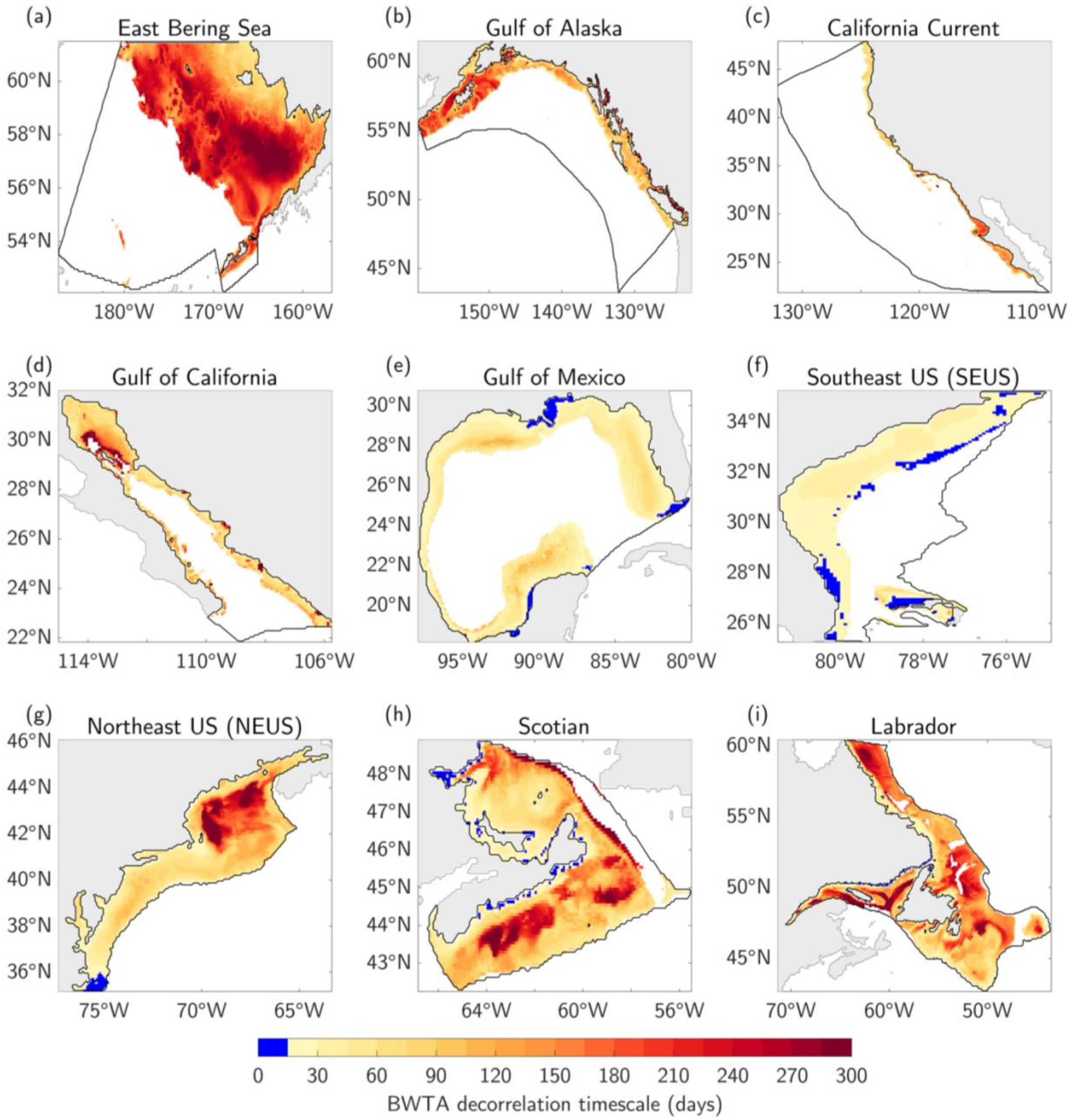

Fig. S5 Decorrelation timescale (days) of daily mean bottom water temperature (BWT) anomalies from 1993-2019. Blue shading denotes decorrelation timescales of less than 15 days.

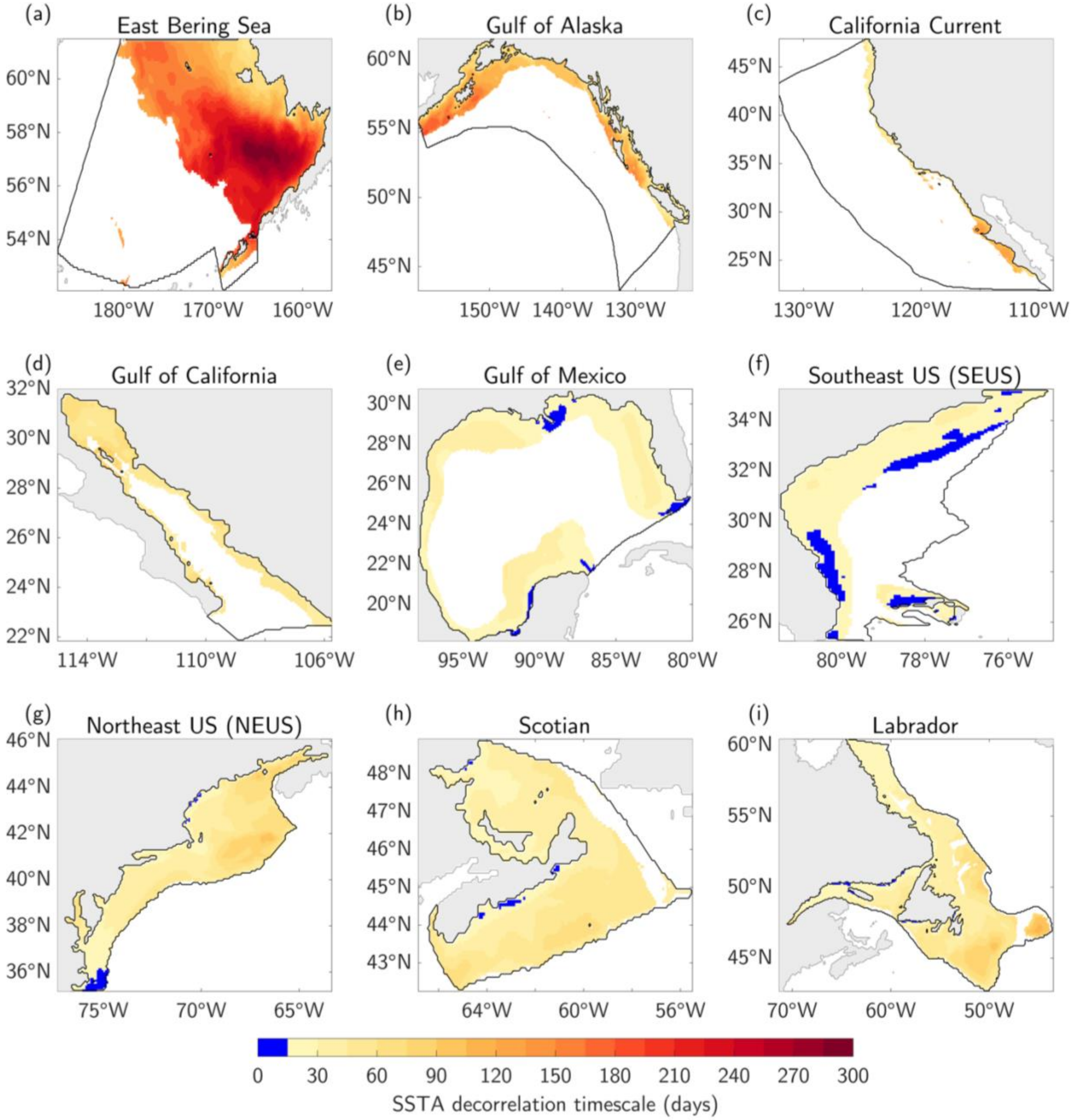

Fig. S6 Same as Fig. S5, but for daily mean SST anomalies.

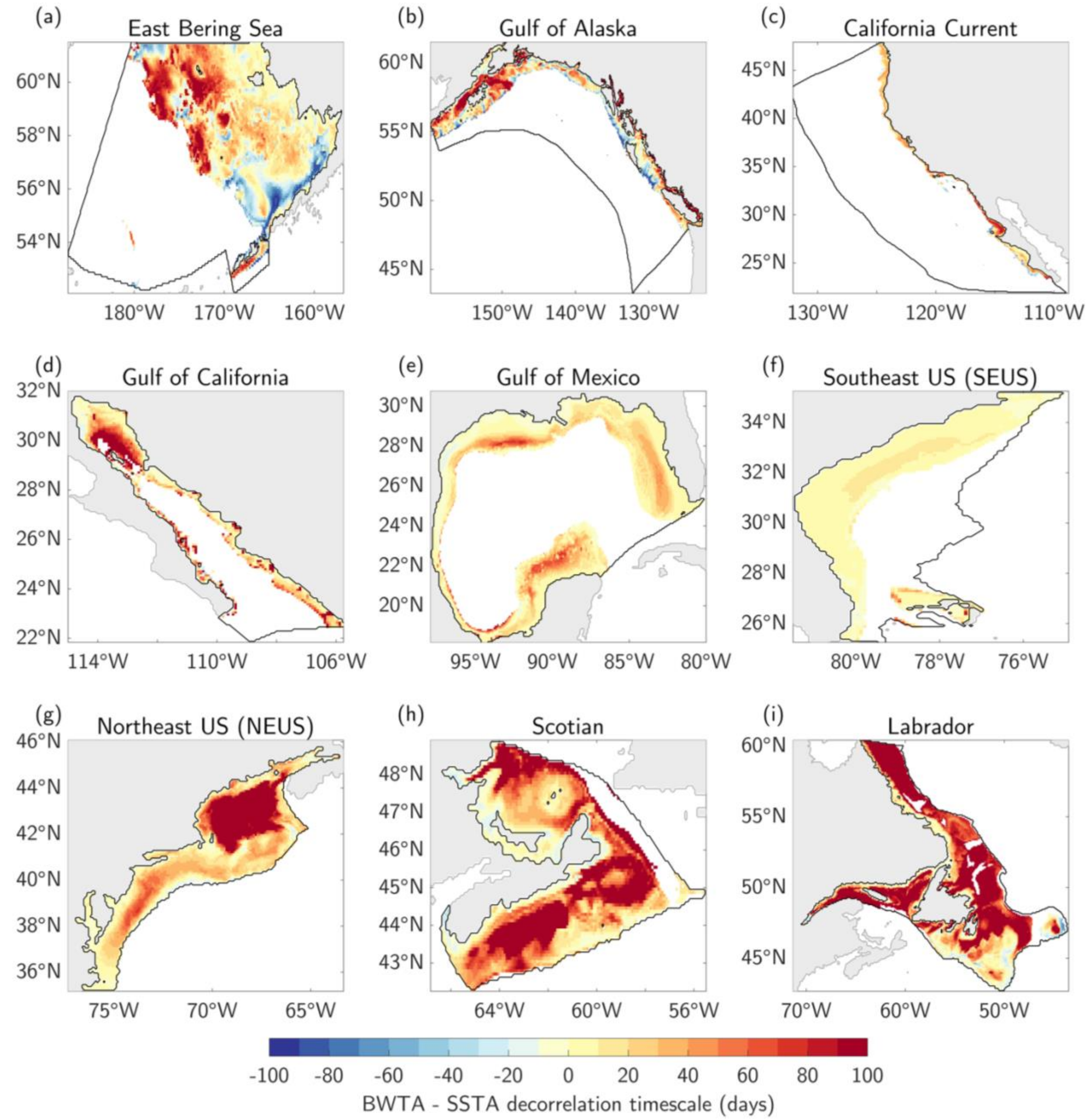

Fig. S7 Difference in decorrelation timescales between daily mean BWT and SST anomalies from 1993-2019.

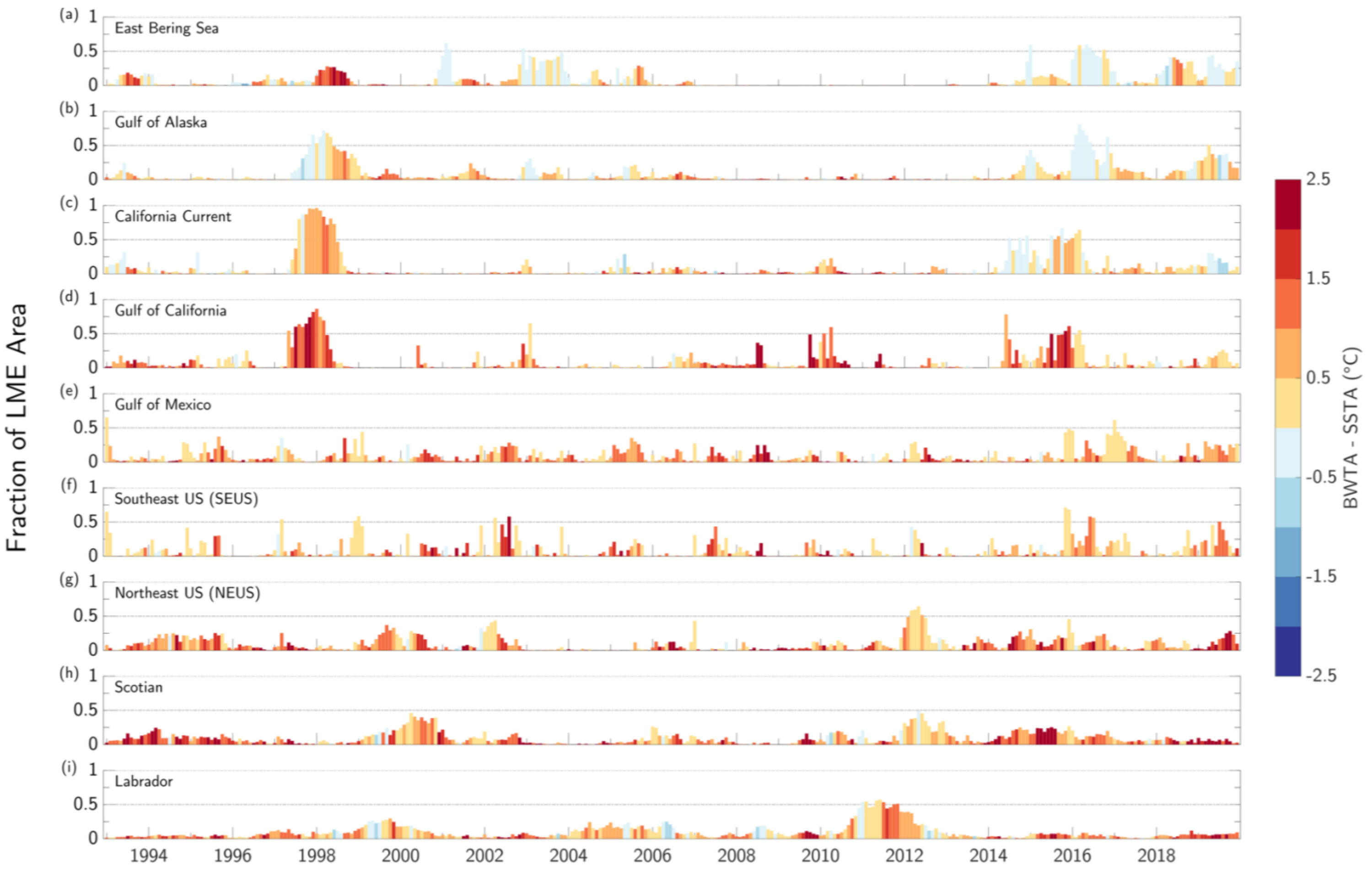

Fig. S8 The percentage of each LME's area experiencing BMHW conditions for each month from 1993-2019. Shading denotes difference of BWT anomalies minus SST anomalies (˚C) averaged across all grid cells experiencing BMHW conditions. Horizontal gray lines mark areal extents of 0.5 and 1. Note only grid cells with bottom depths < 400m were used for areal percentage and intensity difference calculations.

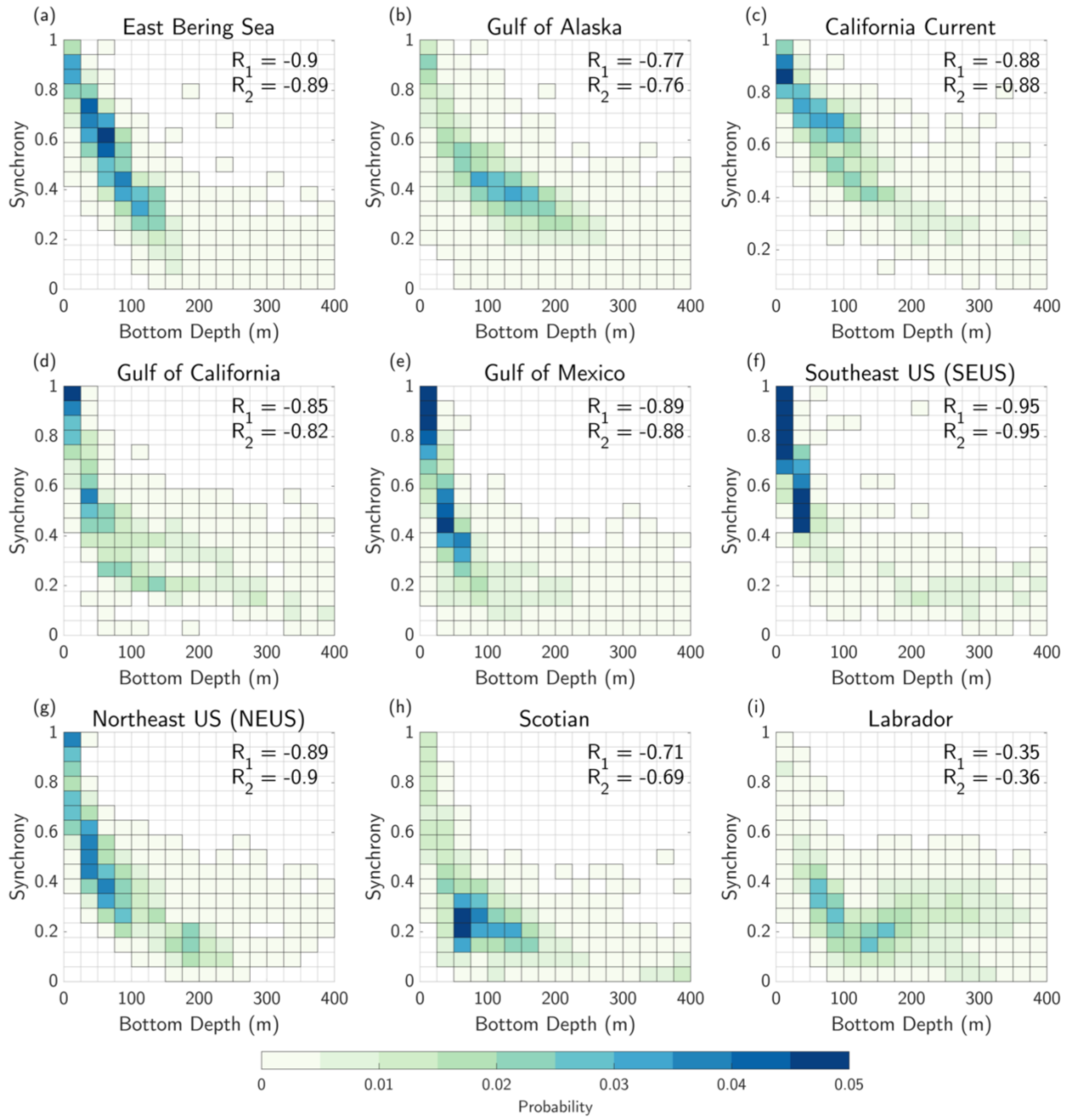

Fig. S9 As in Fig. S2, but for BMHW and SMHW temporal synchrony (i.e., main text Fig. 9) versus bottom depth (m). The Spearman correlations for a 1st- and a 2nd-order polynomial model fit are shown in the top right corner of each panel (R_1 and R_2, respectively).

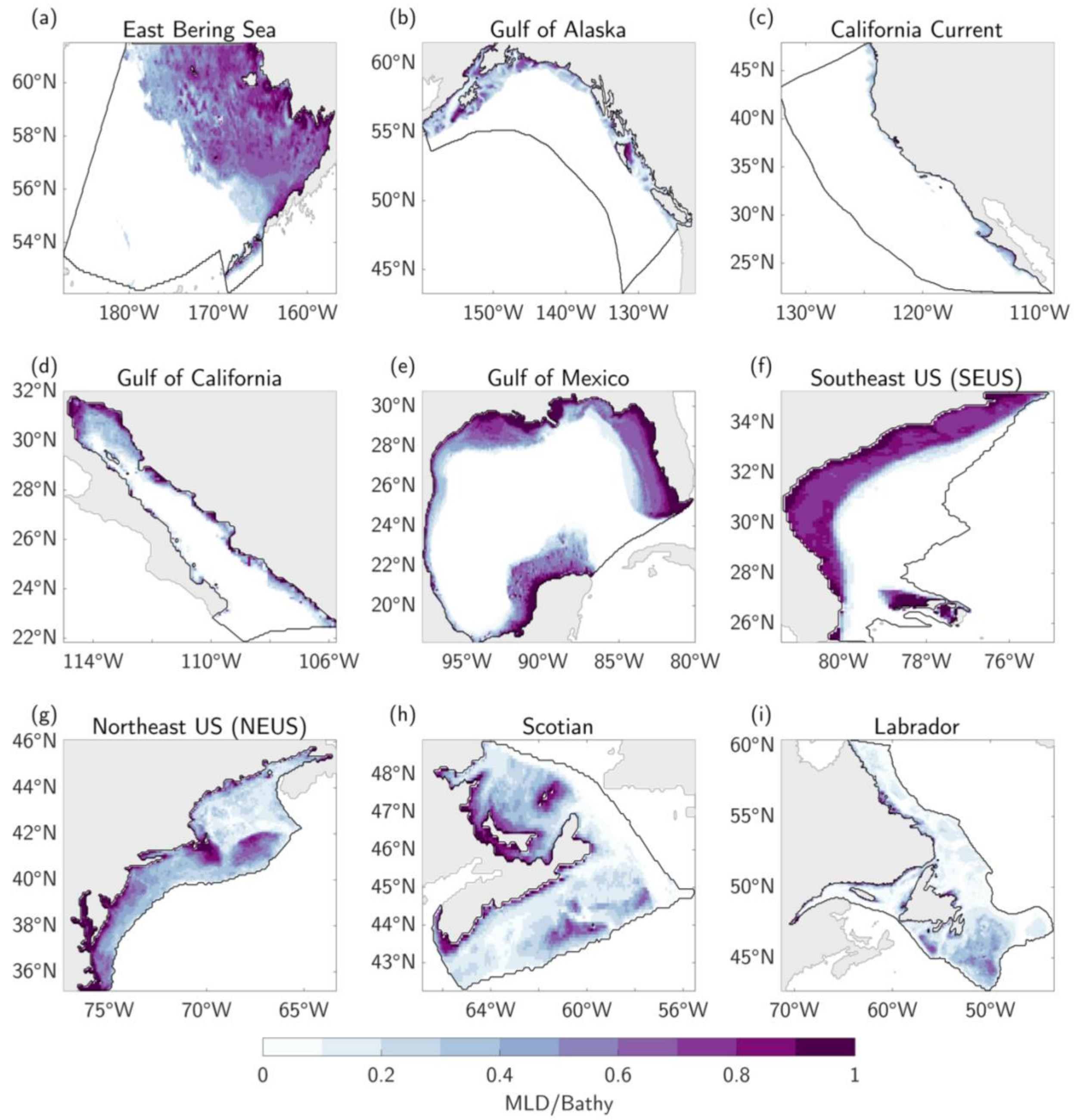

Fig. S10 Mixed layer depth (MLD) divided by bathymetric depth composited at each grid cell when BMHW and SMHW conditions co-occur from 1993-2019. A value of 1 indicates that the MLD reaches the ocean floor.

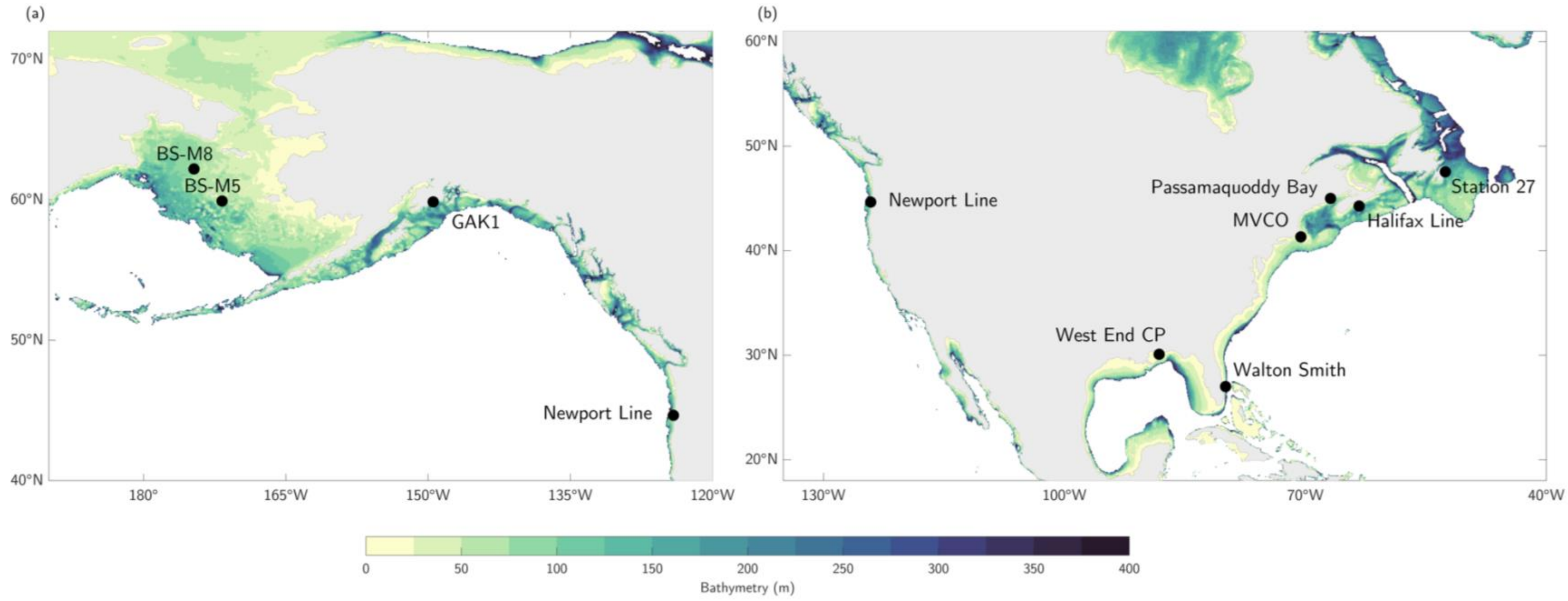

Fig. S11 Location of different long-term near-bottom temperature observations used to compare with GLORYS (see Supplementary Methods). Shading denotes ocean bottom depth (m), as in Fig. 1 of the main text.

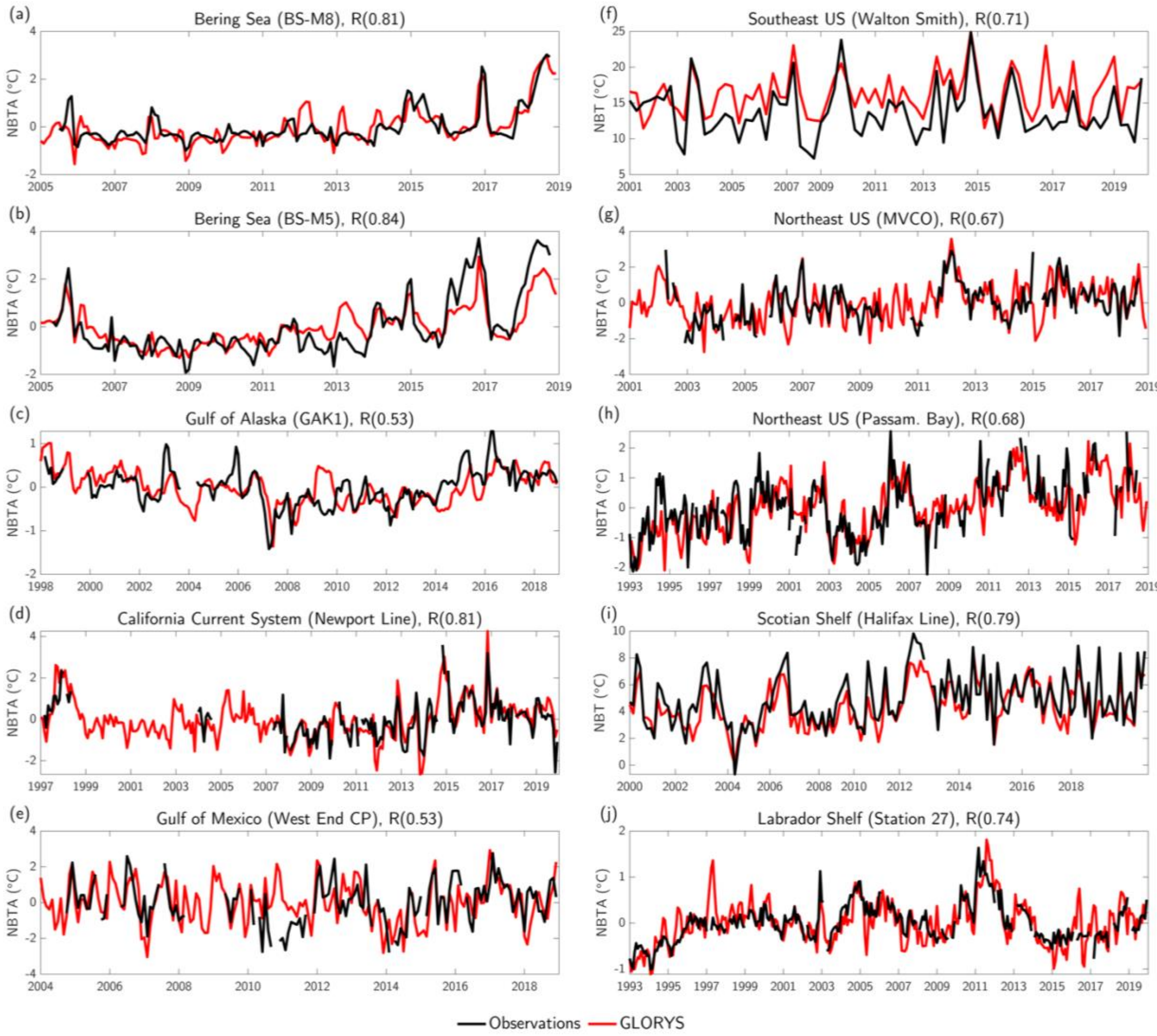

Fig. S12 Timeseries of near-bottom temperature (NBT; ˚C) at the different locations shown in Fig. S11. Observations are in black and GLORYS is in red. All values are monthly mean NBT anomalies except for the Walton Smith and Halifax Line locations, which are based on ~quarterly NBT measurements. The correlation coefficient (R-value) between observations and GLORYS at each location is shown in the title. All R-values are significant at the 99% confidence level.

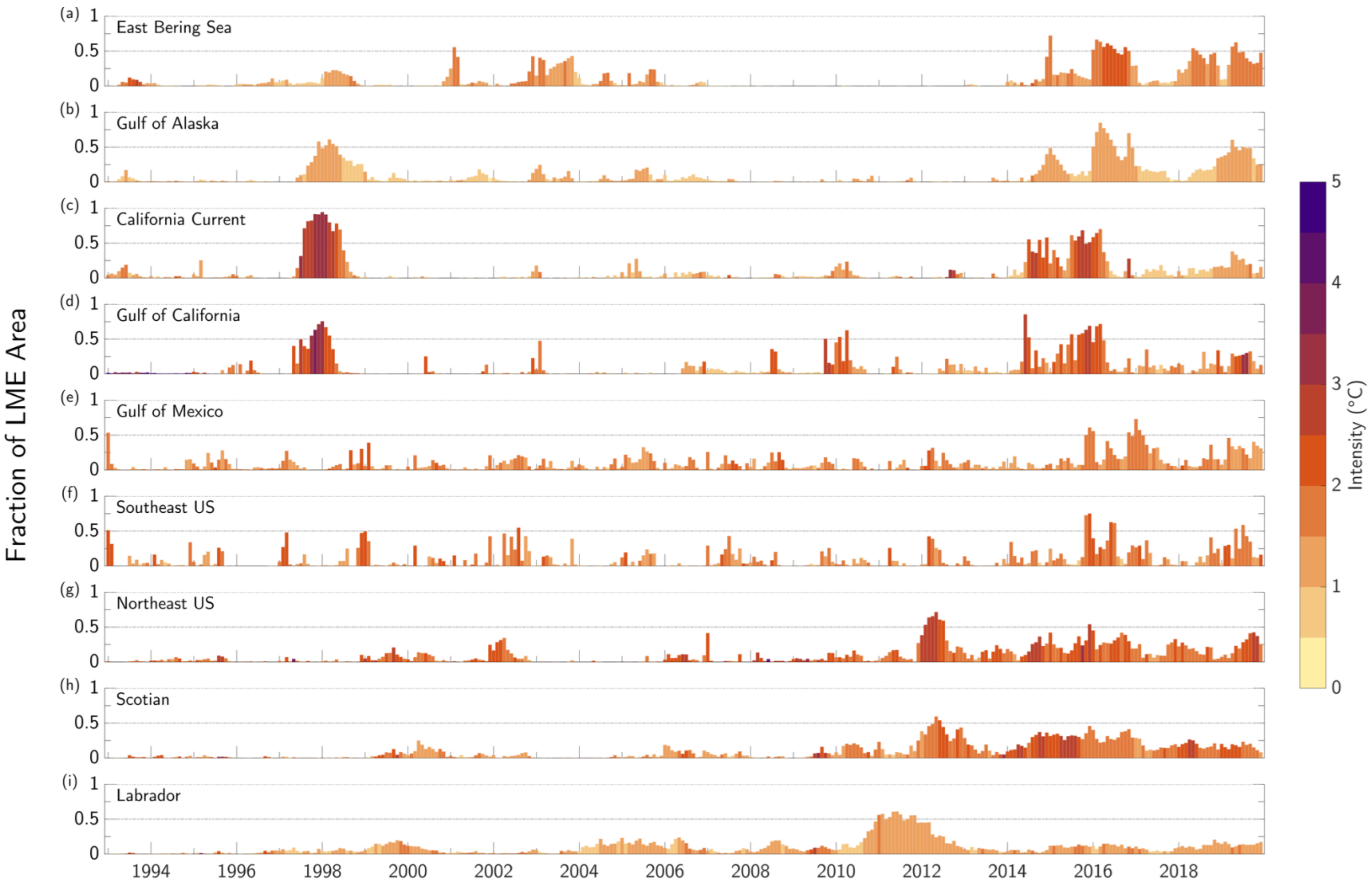

Fig. S13 Same as Fig. 5 of the main text, but for the raw (i.e., not detrended) bottom water temperature data.

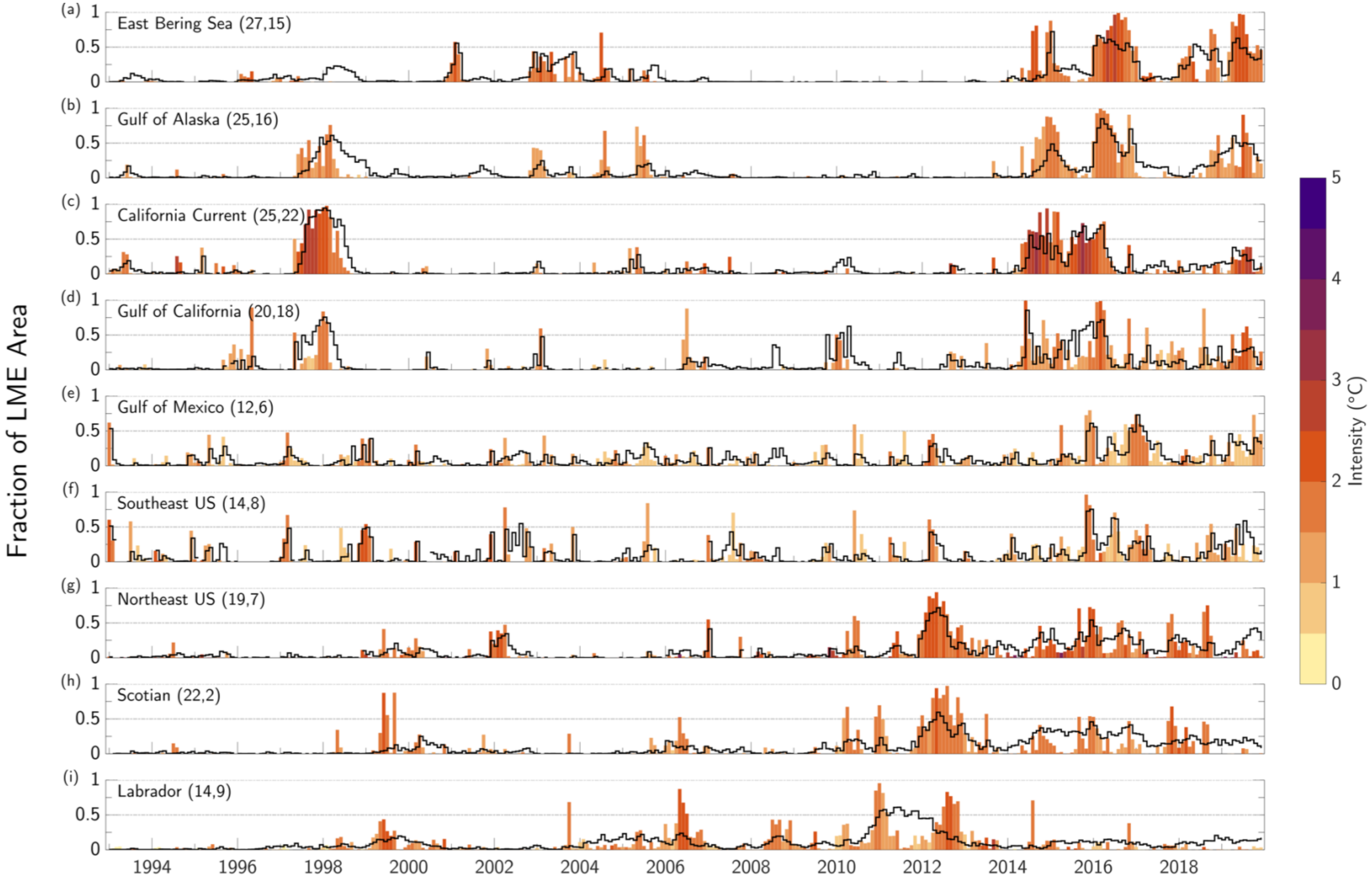

Fig. S14 Same as Fig. 8 of the main text, but for the raw (i.e., not detrended) sea surface and bottom water temperature data.
